# Supplementary material for: Clinical utility of comprehensive genomic profiling in Japan: Result of PROFILE-F study
Source: PLoS One. 2022 Mar 31;17(3):e0266112. doi: 10.1371/journal.pone.0266112 (PMC8970371; doi:10.1371/journal.pone.0266112)
Supplement: Attachment — Submitted filename: comments.docx [file pone.0266112.s002.docx]

Summary Comments

Overall, this study’s goal is to describe the utility of Clinical genomic sequencing in Japan, a country that historically puts significant limitations on who can receive NGS sequencing and, as a consequence, limited data is available from regarding its clinical utility.

I think the data they have collected could be useful to have in the literature, but as of now, the authors present the data purely in an observational format without providing any context or statistical comparisons about how their data in Japanese populations compare to previously published Foundation CDx retrospective studies.

Given the only novelty of this paper would be how numbers of clinically relevant alterations compare in populations in Japan vs other populations, statistical comparisons between this study's population and others are important to include as part of the results in order to provide context.

I’m unsure about the use of the word “Actionability” in the context of their document. I believe the right phrase may instead be “Clinical Utility”. In my thought process, “actionability” would imply an alteration that would change the treatment options available to a patient. If possible, It would be helpful to report which of the alterations reported in Foundation Dx fall under “actionable”. Based on the frequency reported (including an average of 3 “actionable” alterations/patient) I have to assume many of these alterations are only clinically significant in their prognostic abilities, not in their ability to open therapeutic options. Examples of these are TP53 or KRAS, which are clinically useful from a prognostic standpoint, but I feel calling them “actionable” may be disingenuous.

Otherwise, there were some basic English and grammatical errors that were clearly derived from "english as a second language" primary authors. Of course, I recognize how difficult it is to write scientific articles in this context, so I tried to provide some suggested grammatical improvements below, just as a way of improving eventual readability of document.

Abstract

Line 41: A molecular tumor board had been completed on all 130 cases by October 31, 2019. The median number of gene alterations detected by Foundation testing, excluding variants of unknown significance (VUS)) was4 (ranged 0 to 21) per 42 case.

Introduction:

English/Grammatical Changes

Line 49: In recent years, next-generation sequencing (NGS), which is a technology for analyzing the base sequence of DNA in a short time, has made it possible to examine a large amount of genomic information at once.

Line 50: Conventionally, cancer drug therapy has been selected ~~by each~~ based on the organ of the primary tumor.

Line 51: However, it has been found that gene alterations in cancer cells ~~have diversity~~ can effect biology and treatment, even in cancers ~~types~~ originating from the same organ.

Line 55: The CGP tests, FoundationOne® CDx cancer genome profiling and OncoGuide™ NCC Oncopanel System, have been covered by Public health insurance since June 2019 and are widely adapted in Japan.

Line 62: You mention the term “Heme” but I’m not sure what this is in reference to as it has not been previously identified. I think it would be less confusing to state explicitly “Foundation one CDx or Foundation one Heme” at this point

Methods:

English/Grammatical Changes

Line 66: We retrospectively reviewed 130 cases of patients with advanced solid tumors who either progressed on, or were finishing standard systemic therapy.

Line 74: sequencing of 324 genes with FoundationOne … and RNA sequencing of 265 (transcripts ?)

Clarifications/Concerns

Definition of Actionability: I’m not quite sure I agree with the definition presented in the article. “Actionability” suggests that knowing this information will influence the treatment decisions made by the provider. Many of the mutations that fall within (1) likely inform treatment efficacy, but do not change treatment decisions as would not suggest alternative treatment options. I’m wondering if “clinical relevance” may be a more accurate terminology, with “actionability” only being used for alterations that fall under (2). However, I do not feel strongly about this position.

**Results:**

*Patients and characteristics*

*English/Grammatical Changes*

Line 97: I’m not sure its necessary to include all cancers that were present in dataset within the main body of text. Could instead just refer to supplement table, with the frequency of each cancer typ in that table.

Line 117: VUS were contained. This seems like a fragment sentence and I’m not really sure what it is referring to.

*Clarifications/Concerns*

It would be instructive to know the specific numbers of failed Foundation reports, which tumor types they failed in as a supplemental table.

*The common alterations and differences by each cancer type*

The choices for which observations to take from this data and highlight in the results feel very arbitrary, are not given any context within the published literature. Many observational studies from Foundatiaon reports have been published. The goal, and purported novelty, of this paper is to compare these studies with the prevalence of alterations within a clinical population in Japan. Then that’s what the authors should be doing during the results: highlighting any significant prevalence differences between their population and published studies. Even if there are no such statistically significant differences, they should test for differences and report negative results. Finally, I would like to know which alterations they actually had the power to detect a significant difference between their population and previously reported rates, so I know which alterations may still be different within this population if there was a larger sample size to compare. I come away with no increased knowledge about the clinical prevalence of these alterations than I had previously as someone with a general understanding of alteration rates in cancer.

*Alterations and actionability*

*Clarifications/Concerns*

I’ll dispute a little bit the terminology of “actionable”. I would not consider TP53 loss or KRAS mutation “actionable” alterations as they would not lead to a change in treatment decision or additional therapeutic options. Many of these alterations may be “clinically relevant” as they can inform prognosis or treatment efficacy. In that sense, the foundation report may have some clinical utility, but not necessarily “actionability”. I mention this because a rate of 3 actionable targets per tumor seems exceedingly high to report.

Again, the novelty of this research would be the comparison between the Japanese population and previous Foundation CDx report studies. It’s not fair to ask the reader to make these inferences. They authors should report statistical differences between their population and previous reports.

*Secondary findings*

As above, context about rates on hereditary cancer dx from Foundation CDx in non-japanese populations are needed for context.

**Discussion**

First line of discussion: Recently, tumor agonistic genomic medicine, which uses somatic or germline genetic alterations to guide decisions about treatment choice , has been attracting attention.

Third paragraph: “*In Japan, NGSCGP can only be used at the time of completion or expected completion of standardtreatment to receive the reimbursement of Public Health Insurance. Therefore, it is difficult to make simple comparisons with foreign literature, and accumulation of data in Japan is necessary.”*

I actually think including this information within the Introduction may be beneficial to help frame the goals and previous limitations in the current data.

**Figures**

Figure 1B-1G: Can you please title the graphs with which organ class they are representing within the figure as well as the legend. It makes it easier to read the figure.
