# Supplementary material for: Clinical utility of comprehensive genomic profiling in Japan: Result of PROFILE-F study
Source: PLoS One. 2022 Mar 31;17(3):e0266112. doi: 10.1371/journal.pone.0266112 (PMC8970371; doi:10.1371/journal.pone.0266112)
Supplement: Attachment — Submitted filename: Response to Reviewers.docx [file pone.0266112.s003.docx]

**Journal Requirements:**

*When submitting your revision, we need you to address these additional requirements.*

1. *Please ensure that your manuscript meets PLOS ONE's style requirements, including those for file naming. The PLOS ONE style templates can be found at  https://journals.plos.org/plosone/s/file?id=wjVg/PLOSOne_formatting_sample_main_body.pdf and  https://journals.plos.org/plosone/s/file?id=ba62/PLOSOne_formatting_sample_title_authors_affiliations.pdf*

**Reply: I have checked and corrected the above matters.**

*2. We note that the grant information you provided in the ‘Funding Information’ and ‘Financial Disclosure’ sections do not match.*

*When you resubmit, please ensure that you provide the correct grant numbers for the awards you received for your study in the ‘Funding Information’ section.*

**Reply: I confirm and correct the above statement.**

*3. In your Data Availability statement, you have not specified where the minimal data set underlying the results described in your manuscript can be found. PLOS defines a study's minimal data set as the underlying data used to reach the conclusions drawn in the manuscript and any additional data required to replicate the reported study findings in their entirety. All PLOS journals require that the minimal data set be made fully available. For more information about our data policy, please see http://journals.plos.org/plosone/s/data-availability.*

*"Upon re-submitting your revised manuscript, please upload your study’s minimal underlying data set as either Supporting Information files or to a stable, public repository and include the relevant URLs, DOIs, or accession numbers within your revised cover letter. For a list of acceptable repositories, please see http://journals.plos.org/plosone/s/data-availability#loc-recommended-repositories. Any potentially identifying patient information must be fully anonymized.*

*Important: If there are ethical or legal restrictions to sharing your data publicly, please explain these restrictions in detail. Please see our guidelines for more information on what we consider unacceptable restrictions to publicly sharing data: http://journals.plos.org/plosone/s/data-availability#loc-unacceptable-data-access-restrictions. Note that it is not acceptable for the authors to be the sole named individuals responsible for ensuring data access.*

*We will update your Data Availability statement to reflect the information you provide in your cover letter.*

**Reply: I upload our minimal underlying data set as Supporting Information files.**

*4. Please include captions for your Supporting Information files at the end of your manuscript, and update any in-text citations to match accordingly. Please see our Supporting Information guidelines for more information: http://journals.plos.org/plosone/s/supporting-information.*

**Reply: I correct the above matter.**

**Reviewer #1** *The authors reported the clinical utility of CGP test in Japan using commercial targeted sequencing system, and they concluded it might be useful for detecting gene alterations in various cancer types and exploring treatment options.*

**Reply: We thank the Reviewer 1’s favorable comments.**

*Major comments:*

1. *The definition of actionability in this report is ambiguous. Please redefine the actionability using evidence levels in accordance with previously reported guidance.*

**Reply: We thank reviewer’s comment regarding actionability. This is an area of on-going debate. We added Fig 3 showing evidence level defined by C-CAT (The Center for Cancer Genomics and Advanced Therapeutics) and elaborated in discussion.**

2. *Please mention the status of genomic medicine in Japan more exactly. For example, detection rate of actionable genomic alteration and implementation rate of genotype-matched treatment in other platforms such as OncoPrime, P5, PleSSision, CANCERPLEX, CLHURC and so on.*

**Reply: We summarized recent reports of clinical utility of CGP in Japan and commented in discussion.**

3. *VUS should not be contained in Fig1A-G.*

**Reply: We revised the figure excluding VUSs.**

4. *Please mention the limitation of this study.*

**Reply: We thank the reviewer to remind us of including the limitation of the study. We added limitation in discussion.**

5. *The authors concluded that many issues still need to be improved in conclusion of the abstract. However, those many issues are not referred in this paper. Please discuss the problems of genomic medicine in Japan more.*

**Reply: We thank this reviewer for his remarks. We added the problems of genomic medicine in Japan in discussion.**

6. *Please attach all genomic information data of the patients in Supplement files.*

**Reply: We added Supporting Information files that includes key genomic data used in this study.**

*Minor comments:*

1. *Please classify ‘Gastrointestinal cancer’, ‘Hepatic Biliary and Pancreatic cancer’, ‘Lung Cancer,’ ‘Gynecologic cancer’ into esophagus, stomach, colorectal, liver, biliary, pancreas, NSCLC, SCLC, ovary, uterus.*

**Reply: Thank you for your suggestion. We revised figures with more detailed classifications.**

2. *Patient No.19 should be removed from the lists of patients who received the treatment corresponding to the gene alteration in Table 3. This is not the case of genotype-matched treatment.*

**Reply: Thank you for your comment. We removed the patient No. 19 from the table 3 and revised result and discussion sections.**

3. *What is the definition of TMB-High in this report?*

**Reply: TMB more than 10 muts/Mb is defined as TMB-high. In case No. 19, the patient had TMB 9 muts/Mb (intermediate), but since there were no other genetic alterations to target for treatment, after discussion among the experts in the field, it was decided to target TMB for treatment. Case No. 24 showed a mutation in the MSH6 gene. The TMB was 9, which is not in the TMB-High category by definition, but together with the MSH6 mutation, it was considered to be a target for treatment. Therefore, we have modified the expression TMB-High in Table 3, No.19 and No.24.**

**Reviewer #2**

*Overall, this study’s goal is to describe the utility of Clinical genomic sequencing in Japan, a country that historically puts significant limitations on who can receive NGS sequencing. As a consequence, limited data is available from this country regarding NGS clinical utility.*

*I think the data they have collected could be useful to have in the literature, but as of now, the authors present the data purely in an observational format without providing any context or statistical comparisons about how their data in Japanese populations compare to previously published Foundation CDx retrospective studies.*

*Given the only novelty of this paper would be how numbers of clinically relevant alterations compare in populations in Japan vs other populations, statistical comparisons between this study's population and others are important to include as part of the results in order to provide context.*

**Reply: Thank you for your suggestion. We tried statistical comparison to foreign literatures. But it was difficult. We added this in limitation.**

*I’m unsure about the use of the word “Actionability” in the context of their document. I believe the right phrase may instead be “Clinical Utility”. In my thought process, “actionability” would imply an alteration that would change the treatment options available to a patient. If possible, It would be helpful to report which of the alterations reported in Foundation Dx fall under “actionable”.*

*Based on the frequency reported (including an average of 3 “actionable” alterations/patient) I have to assume many of these alterations are only clinically significant in their prognostic abilities, not in their ability to open therapeutic options. Examples of these are TP53 or KRAS, which are clinically useful from a prognostic standpoint, but I feel calling them “actionable” may be disingenuous. However, I do not feel strongly about this semantic distinction if it doesn't bother the editor or other reviewers.*

**Reply: Thank you for your comments. We explain the definition of actionable in discussion.**

*From a recommendation standpoint, if they are able to provide statistical context for how the results in their population compare with previous Foundation CDx reports in USA and Europe, including alteration prevalence and power to detect statistical differences, I think the paper is worth adding to the literature via PLOSOne publication. (more detailed comments in the attached document).*

**Reply: Thank you for your suggestion. We tried statistical comparison to foreign literatures. But it was difficult. We added it in limitation.**

**We also thank the review #2 for suggested changes in the manuscripts. We included these suggestions in the revised manuscript.**
